# Supplementary material for: Utilising Digital Health Technology to Support Patient-Healthcare Provider Communication in Fragility Fracture Recovery: Systematic Review and Meta-Analysis
Source: Int J Environ Res Public Health. 2019 Oct 22;16(20):4047. doi: 10.3390/ijerph16204047 (PMC6843966; doi:10.3390/ijerph16204047)
Supplement: Supplementary file 1 [file ijerph-16-04047-s001.zip › IJERPH_22Oct19_577911 Supplementary_1_logic grid.docx]

| **Fractures** | **Older adults** | **Models of care/approach** | **Education/patient engagement** |
| --- | --- | --- | --- |
| Fractures, bone[mh]  Hip fractures[mh]  Spinal fractures[mh]  Hip fracture*[tiab]  Osteoporotic fractures[mh]  Osteoporosis[mh]  Fragility fracture*[tiab]  Proximal femur fracture*[tiab]  Intertrochanteric fracture[tiab]  Intracapsular fracture[tiab] | Frail elderly[mh]  Elder*[tiab]  Older*[tiab]  Aged*[tiab]  Frail*[tiab]  Frailty[mh]  Geriatrics[mh]  Multimorbidity[mh] | Rehabilitation[mh]  Recovery[tiab]  Post discharge[tiab]  Telemedicine[mh]  eHealth[tiab]  mHealth[tiab]  Orthogeriatric[tiab]  Post hospital[tiab]  Post acute[tiab]  Multidisciplinary[tiab]  Integrated care[tiab]  Health and social care[tiab]  Physiotherapy[tiab]  Physical therapy modalities[mh]  Patient discharge[mh]  Patient-centered care[mh]  Extended care[tiab]  Health restructuring[tiab]  Health systems[tiab]  Delivery of health care, integrated[mh]  Information and communication technologies[tiab]  Patient care[mh]  Continuity of care[mh]  Patient care management[mh]  Models, nursing[mh]  Models, education[mh]  Artificial intelligence[mh]  Health policy[mh] | Education[mh]  Patient participation[mh]  Empowerment[tiab]  Power(psychological)[mh]  Patient empowerment[tiab]  Community empowerment[tiab]  Health literacy[tiab]  Self-management[tiab]  Self-management[mh] (just indexed as MeSH term in 2018)  Learning management system[tiab]  eLiteracy[tiab]  Transformative education[tiab]  Patient education as topic[mh] |

**Database search**

| SR NO | Search terms | Articles resulted |
| --- | --- | --- |
| PUBMED | | |
| 1 | Hip fractures[mh]AND frail elderly[mh] AND rehabilitation[mh] | 46 |
| 2 | Hip fracture*[tiab] AND older*[tiab] AND discharge*[tiab] AND rehab*[tiab] | 189 |
| 3 | Education[mh] AND hip fractures[mh] AND rehabilitation[mh] | 41 |
| 4 | Power (psychology)[mh] AND hip fractures[mh] AND rehabilitation[mh]  (here power corresponding to empowerment) | 1 |
| 5 | Health literacy[tiab] AND hip fracture*[tiab] AND recovery*[tiab] | 1 |
| 6 | Community*[tiab] AND hip fracture*[tiab] AND recovery*[tiab] | 121 |
| 7 | Self-management [tiab] AND hip fracture*[tiab] AND recovery*[tiab] | 1 |
| 8 | Recovery[tiab] AND hip fractures[tiab] | 217 |
| 9 | Recovery[tiab] AND hip fractures[tiab] AND post discharge[tiab] | 2 |
| 10 | Orthogeriatric[tiab] AND hip fractures [tiab] | 76 |
| 11 | Physiotherapy[tiab] AND hip fractures[tiab] | 26 |
| 12 | Patient discharge[mh] AND integrated care[tiab] AND hip fracture[tiab] | 1 |
| 13 | Patient discharge[mh] AND multidisciplinary[tiab] AND hip fracture[tiab] | 14 |
| 14 | Fragility[tiab] AND fractures[tiab] AND frail elderly[mh] | 55 |
| 15 | Fragility[tiab] AND fractures[tiab] AND frail elderly[mh] AND patient discharge[mh] | 3 |
| 16 | Patient centered care[mh] AND patient discharge[mh] AND fracture*[tiab] | 4 |
| 17 | Healthcare restructuring[tiab] | 34 |
| 18 | Integrated care[tiab] AND ehealth[tiab] | 22 |
| 19 | Ehealth[tiab] AND “health systems”[tiab] | 79 |
| 20 | Delivery of health care, integrated[mh] AND telemedicine[mh] | 232 |
| 21 | Eliteracy[tiab] | 1 |
| 22 | Patient participation[mh] AND Fractures, bone[mh] | 41 |
| 23 | Patient participation[mh] AND fractures, bone[mh] AND frail elderly[mh] | 1 |
| 24 | Fractures, bone[mh] AND frail elderly[mh] | 388 |
| 25 | Fractures, bone[mh] AND frail elderly[mh] AND patient discharge[mh] | 9 |
| 26 | Fractures, bone[mh] AND frail elderly[mh] AND patient discharge[mh] AND Patient-centered care[mh] | 2 |
| 27 | Mellick Chehade[Author] | 18 |
| 28 | Tiffany Gill[Author] | 99 |
| 29 | Lalit Yadav[Author] | 7 |
| 30 | Renuka Visvanathan[Author] | 90 |
| 31 | Morley JE[Author] AND fractures, bone[mh] | 16 |
| 32 | Mohit Bhandari[Author] AND fragility fracture*[tiab] | 7 |
| 33 | Fragility fracture*[tiab] AND integrated care[tiab] | 2 |
| 34 | Fragility fracture*[tiab] AND persanali* care[tiab] | 1 |
| 35 | Fragility fracture*[tiab] AND self management[tiab] | 3 |
| 36 | Fragility fracture*[tiab] AND education*[tiab] | 82 |
| 37 | Osteoporosis[tiab] AND telemedicine[tiab] | 11 |
| 38 | Osteoporosis[mh] AND telemedicine[mh] | 8 |
| 39 | Hip fractures[mh] AND telemedicine[mh] | 2 |
| 40 | Osteoporosis[mh] AND eHealth[tiab] | 1 |
| 41 | Frailty[tiab] AND telemedicine[tiab] | 7 |
| 42 | Transformative* education*[tiab] AND patient care[mh] | 27 |
| 43 | Transformative* education*[tiab] AND continuity of patient care[mh] | 10 |
| 44 | Self-management[mh] AND continuity of patient care[mh] | 49 |
| 45 | Osteoporosis[mh] AND models, nursing[mh] | 5 |
| 46 | Osteoporosis[mh] AND models, nursing[mh] AND continuity of patient care[mh] | 1 |
| 47 | Fractures, bone[mh] AND models, nursing[mh] | 14 |
| 48 | Patient education as topic[mh] AND telemedicine[mh] | 727 |
| 49 | Patient education as topic[mh] AND telemedicine[mh] AND osteoporosis[mh] | 2 |
| 50 | Artificial intelligence[mh] AND osteoporosis[mh] | 56 |
| 51 | Artificial intelligence[mh] AND geriatrics[mh] | 28 |
| 52 | Artificial intelligence[mh] AND frailty[mh] | 2 |
| 53 | Artificial intelligence[mh] AND fragility fractures[tiab] | 1 |
| 54 | Fractures, bone[mh] AND multimorbidity[mh] | 3 |
| 55 | Health policy[mh] AND frailty[mh] | 2 |
| 56 | Telemedicine[tiab] AND fragility fractures[tiab]  Telehealth[tiab] AND fragility fractures[tiab]  Mhealth[tiab] AND fragility fractures[tiab]  Behaviour change[tiab] AND fragility fractures[tiab] | 0 |
| CINAHL | | |
| 56 | Telemedicine or telehealth or telecare [AB] AND osteoporosis [AB] AND patient education [AB} | 2 |
| Embase | | |
| 57 | Telehealth[tiab] AND osteoporosis [tiab] AND patient education [tiab] | 1 |
| ProQuest dissertations and thesis global | | |
| 58 | “fragility fractures” AND “Integrated care” | 4 |
| 59 | “fragility fractures” | 441 |
| Scopus | | |
| 60 | Osteoporosis AND telemedicine[title/abstract/keywords) | 32 |
| 61 | Osteoporosis AND eHealth[title/abstract/keywords) | 7 |
| 62 | “fragility fracture” AND “information and communication technologies” | 1 |
| Google scholar | | |
| 50 | “fragility fracture” AND eHealth | 89 |
| Total | | 3465 |
| Considered for title and abstract screening- removing all duplicates and articles with no clear orthopaedics or fragility/osteoporotic fracture domain | | 1686 |
| Considered for full text review | | 38 |
| Included for systematic review | | 11 |
| Additional articles included from manual search/secondary references | | 4 |
| Total articles included in this review | | 15 |
